# Optimization of Culture Conditions for Secretory Production of 3-Hydroxybutyrate Oligomers Using Recombinant *Escherichia coli*

**DOI:** 10.3389/fbioe.2022.829134

**Published:** 2022-02-25

**Authors:** Tetsuo Sakurai, Shoji Mizuno, Yuki Miyahara, Ayaka Hiroe, Seiichi Taguchi, Takeharu Tsuge

**Affiliations:** ^1^ Department of Materials Science and Engineering, Tokyo Institute of Technology, Yokohama, Japan; ^2^ MIRAI, Japan Science and Technology Agency (JST), Saitama, Japan; ^3^ Department of Chemistry for Life Sciences and Agriculture, Faculty of Life Sciences, Tokyo University of Agriculture, Setagaya, Japan

**Keywords:** class IV PHA synthase, oligomer, secretion, fed-batch culture, alcoholysis, chain transfer reaction, 3-hydroxybutyrate

## Abstract

Poly(3-hydroxybutyrate) [P(3HB)] is the most representative polyhydroxyalkanoate (PHA), which is a storage polyester for prokaryotic cells. P(3HB)-producing recombinant *Escherichia coli* secretes diethylene glycol (DEG)-terminated 3HB oligomers (3HBO-DEG) through a PHA synthase-mediated chain transfer and alcoholysis reactions with externally added DEG. The purpose of this study was to optimize the culture conditions for the secretory production of 3HBO-DEG with jar fermenters. First, the effects of culture conditions, such as agitation speed, culture temperature, culture pH, and medium composition on 3HBO-DEG production, were investigated in a batch culture using 250-ml mini jar fermenters. Based on the best culture conditions, a fed-batch culture was conducted by feeding glucose to further increase the 3HBO-DEG titer. Consequently, the optimized culture conditions were reproduced using a 2-L jar fermenter. This study successfully demonstrates a high titer of 3HBO-DEG, up to 34.8 g/L, by optimizing the culture conditions, showing the feasibility of a new synthetic strategy for PHA-based materials by combining secretory oligomer production and subsequent chemical reaction.

## Introduction

Polyhydroxyalkanoate (PHA) is a thermoplastic polyester that accumulates in microbial cells as an energy storage substance from renewable biomasses such as sugars and vegetable oils (Sudesh et al., 2000). Utilizing PHA as an alternative material to petroleum plastics is attracting considerable attention; this is because petroleum plastics are non-biodegradable in nature and cause environmental pollution and ecosystem destruction. PHA, which is microbially produced, has excellent biodegradability in various environments, including marine environments (Suzuki et al., 2021), making it an attractive material for many scientists and engineers. PHAs are promising materials for ensuring sustainability and renewability (Sivashankari et al., 2021; Taguchi and Matsumoto, 2021).

Poly(3-hydroxybutyrate) [P(3HB)], which is one of the most representative PHAs, has high strength and a high melting point that is comparable to polypropylene (Sudesh et al., 2000). However, owing to its brittleness and high production cost, P(3HB) is difficult to use as a commodity plastic material. Numerous studies have been conducted to make P(3HB) a more practical material through copolymerization. The brittleness of P(3HB) can be improved by introducing second monomer units such as 3-hydroxyvalerate, 3-hydroxyhexanoate, 3-hydroxy-4-methylvalerate, and 4-hydroxybutyrate (Sudesh et al., 2000; Tanadchangsaeng et al., 2009). The presence of these second monomer units in the P(3HB) sequence prevents the crystallization of P(3HB), thereby resulting in increased flexibility of the material. Although copolymerization can improve these mechanical properties, it does not contribute to reducing the production cost of PHA.

P(3HB)-producing recombinant *Escherichia coli* secretes a small amount of 3HB oligomers (3HBO) through a PHA synthase-mediated chain transfer (CT) reaction (Miyahara et al., 2019; Hiroe et al., 2021). The CT reaction is induced *via* endogenous ethanol, and elongating polymer/oligomer chains are released by the alcoholysis of thioester bonds between PHA synthase-polymer/oligomer complexes (Hiroe et al., 2013; Tsuge, 2016). Thus, ethanol is conjugated to the carboxyl terminal of 3HBO by functioning as a CT agent.

By contrast, the externally and abundantly added alcohol compounds such as ethanol and ethylene glycol also function as CT agents for PHA synthesis (Utsunomia et al., 2017a; Mizuno et al., 2021; Nduko and Taguchi, 2021). Among the alcohol compounds tested so far, the externally added diethylene glycol (DEG) showed the highest efficacy as a CT agent, resulting in the secretory production of DEG-terminated 3HBO (3HBO-DEG) with a titer of 4.32 g/L (Hiroe et al., 2021). The 3HBO-DEG purified from the culture supernatant can be used as a macromonomer for the synthesis of 3HBO-based polyurethane by reacting with diisocyanate (Hiroe et al., 2021). The 3HBO-based polyurethane is an amorphous polymer with a high transparency, and can counteract the brittleness of P(3HB). Thus, microbial 3HBO-DEG has a great potential for usage in the synthesis of novel 3HBO-based polymers.

Secretory production of 3HBO is a cost-effective approach when compared with conventional intracellular P(3HB) production, as 3HBO can be easily purified from the culture supernatant *via* the solvent extraction method (Hiroe et al., 2021). However, to further enhance the cost-effectiveness of secretory production, it is necessary to increase the titer of 3HBO in the culture broth.

The purpose of this study was to optimize the culture conditions of *E. coli* BW25113*ΔadhE* harboring pGEM-*phaRC*_YB4_ *AB* for the secretory production of 3HBO-DEG with jar fermenters. First, the effects of culture conditions, such as agitation speed, culture temperature, culture pH, and medium composition, on 3HBO-DEG production were investigated in a batch culture using 250-ml mini jar fermenters. Subsequently, a fed-batch culture was conducted by feeding glucose to further increase the 3HBO-DEG titer. Consequently, the optimized culture conditions were reproduced using a 2-L jar fermenter. This study successfully demonstrates the production of high 3HBO-DEG tires up to 34.8 g/L by optimizing culture conditions.

## Materials and Methods

### Bacterial Strains and Plasmids

**FIGURE 1:**
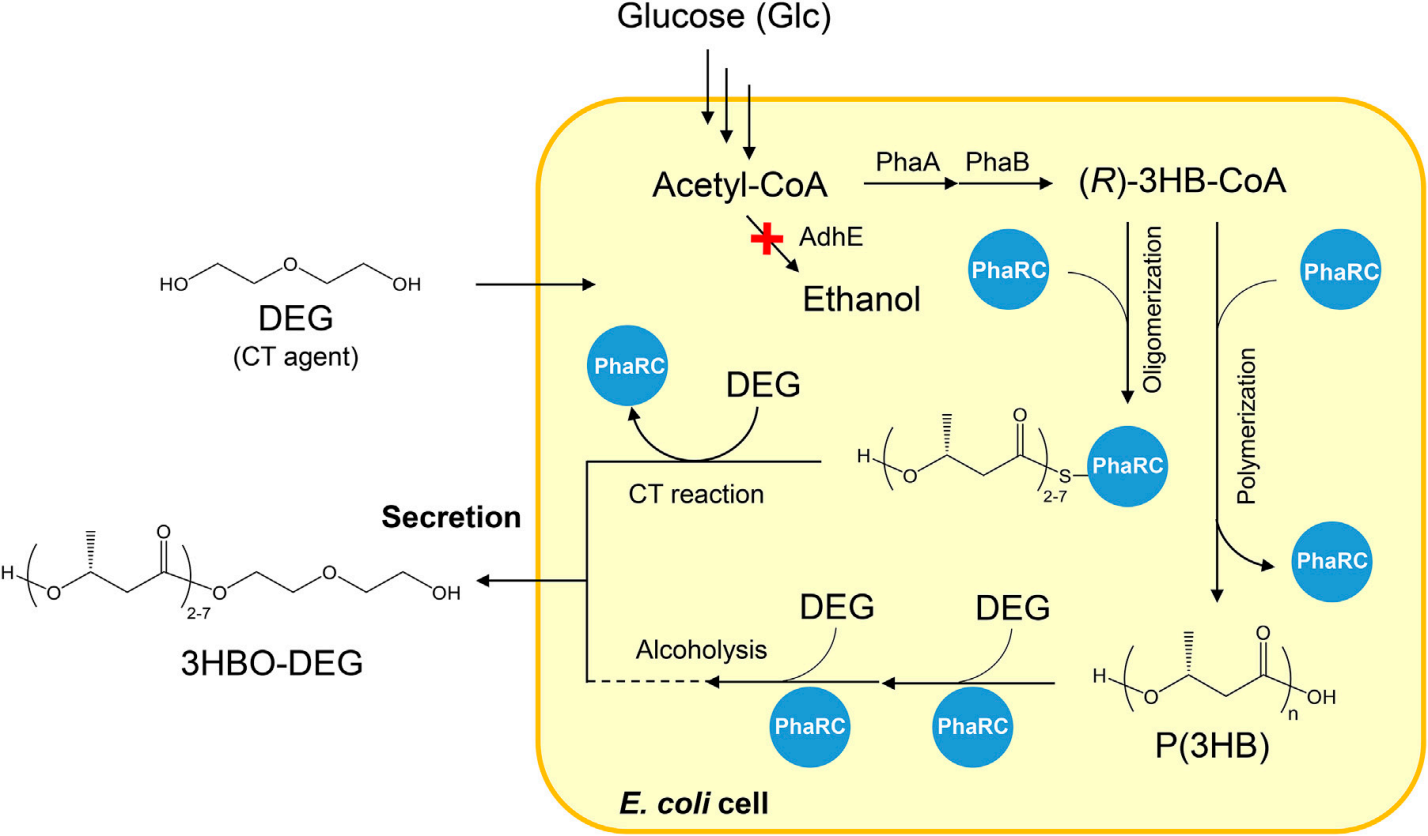
Secretary production of 3HBO-DEG *via E. coli* BW25113Δ*adhE* harboring pGEM-*phaRC*_YB4_ *AB*. AdhE: alcohol dehydrogenase, PhaA: 3-ketothiolase from *R. eutropha* H16, PhaB: acetoacetyl-CoA reductase from *R. eutropha* H16, PhaRC: PHA synthase from *B. cereus* YB-4, 3HB: 3-hydroxybutyrate, DEG: diethylene glycol, CT: chain transfer.

*E. coli* BW25113Δ*adhE* (JW1228), an alcohol dehydrogenase gene (*adhE*)-disrupted strain, was used as the host strain for oligomer production throughout the study. This strain was provided by the National BioResource Project (NBRP), Japan, from the Keio Collection (Baba et al., 2006). The plasmid pGEM-*phaRC*_YB4_ *AB* carrying PHA synthase genes (*phaRC*_YB4_ (Tomizawa et al., 2011)), a 3-ketothiolase gene (*phaA* from *Ralstonia eutropha* H16 (Pohlmann et al., 2006)), and an acetoacetyl-CoA reductase gene (*phaB* from *R. eutropha* H16 (Pohlmann et al., 2006)) were used for 3HBO-DEG production. The synthesis pathway of 3HBO-DEG is shown in Figure 1.

### Batch Culture for 3HBO-DEG Production

For pre-culturing, the recombinant *E. coli* BW25113Δ*adhE* harboring pGEM-*phaRC*_YB4_ *AB* was grown in a Lysogeny Broth (LB) medium (10 g/L Bacto tryptone, 5 g/L Bacto yeast extract, 10 g/L NaCl) at 30°C overnight. The seed culture was inoculated at 1% (v/v) of the initial working volume into a 250-ml jar fermenter (Bio Jr8, Able Corp., Tokyo, Japan) with 100 ml LB medium containing 40 g/L glucose (Glc) and 50 g/L DEG (Kanto Chem. Co., Inc., Tokyo, Japan). To maintain the plasmid pGEM-*phaRC*_YB4_ *AB* in the cells, ampicillin (100 mg/L) was added to the medium. A 500 μl of 1% (w/v) antifoam 204 (Sigma-Aldrich, St. Louis, USA) solution was added into the initial culture medium to prevent the foam generation during cultivation.

To optimize the fermentation conditions, the effects of the agitation speed (150–1,200 rpm), culture temperature (26–36°C), culture pH (pH 5-8 or non-control), NaCl concentration (10–50 g/L in LB medium), type of medium [LB, TB, MR (Y1), and MR (Y5) media] on 3HBO-DEG production were investigated in batch culture operations. The medium composition, other than the LB medium, was as follows: The Terrific broth (TB) medium (Iliev et al., 2018) contained 12 g/L tryptone, 24 g/L yeast extract, 9.4 g/L K_2_HPO_4_, and 2.2 g/L KH_2_PO_4_ dissolved in water. The modified R (MR) medium plus yeast extract [MR (Y1) or MR (Y5) medium] contained 13.5 g/L KH_2_PO_4_, 4 g/L (NH_4_)_2_HPO_4_, 1.4 g/L MgSO_4_ ⋅ 7H_2_O, 1.7 g/L citric acid, Bacto yeast extract (1 g/L or 5 g/L), and 10 ml/L trace metal solution dissolved in water (pH 7.0) (Mohd Fadzil et al., 2018; Mizuno et al., 2021). The trace metal solution contained 10 g/L FeSO_4_·7H_2_O, 2 g/L CaCl_2_, 2.2 g/L ZnSO_4_·7H_2_O, 0.5 g/L MnSO_4_·4H_2_O, 1 g/L CuSO_4_·5H_2_O, 0.1 g/L (NH_4_)_6_Mo_7_O_24_·4H_2_O and 0.02 g/L Na_2_B_4_O_7_·10H_2_O dissolved in 0.1 M HCl. For the pH-stat culture, 1 N NaOH or 1 N HCl was added automatically.

### Fed-Batch Culture for 3HBO-DEG Production

To enhance 3HBO-DEG production, fed-batch cultures with a 250-ml mini jar fermenter were conducted by feeding glucose at a constant rate of 1.25 g-Glc/(L∙h) after 24 h of cultivation using a peristaltic pump SMP-21S (EYELA, Tokyo, Japan). For fed-batch cultures with TB and MR (Y5) media, the glucose feeding rate was temporarily changed to 2.5 g-Glc/(L∙h) depending on the glucose consumption rate. A feed solution composed of 500 g/L glucose, 15 g/L MgSO_4_･7H_2_O, and 0.25 g/L thiamine hydrochloride was used. The fed-batch cultures were performed at 100 ml of the initial working volume, 100 ml/min of air flow (1 vvm), 600 rpm of agitation speed, and a culture temperature of 30°C without pH control.

The fed-batch culture was scaled up using a 2-L scale jar fermenter (Bioneer MDL-8C, B.E. Marubishi, Tokyo, Japan). A 1 L of LB medium supplemented with glucose (40 g/L) and DEG (50 g/L) was used as an initial culture. The feed solution was manually fed at regular intervals to maintain low glucose concentrations. A feed solution composed of 700 g/L glucose, 15 g/L MgSO_4_･7H_2_O, and 0.25 g/L thiamine hydrochloride was used (Wang and Lee, 1997; Kahar et al., 2005). The culture pH was not controlled, and the air flow was set at 1 L/min (1 vvm). Unless otherwise indicated, culture conditions were the same as that of the 250-ml scale mini jar culture.

### Analytical Methods

Following cultivation, the cells were collected *via* centrifugation (5,960 × g, 10 min, 4°C), washed twice with pure water to remove the remaining culture medium components, and then lyophilized. The culture supernatant was collected for 3HBO and 3HB monomer measurements.

Extracellular 3HBO was quantified using the culture supernatant. The culture supernatant of recombinant *E. coli* strain was analyzed before and after acid hydrolysis (Hiroe et al., 2021). One hundred μl of 4 M HCl was added to 100 μL of the culture supernatant and incubated at 100°C for 2 h to hydrolyze the oligomeric 3HB component, after which the reaction mixture was properly neutralized with NaOH solution. The amount of 3HB in the culture supernatant was measured using an enzyme assay with D-3HB enzyme assay kits (J.K. International, Tokyo, Japan). Therefore, the 3HB-DEG titer was described as volumetric weight excluding DEG in this study.

The intracellular P(3HB) content of the dried cells was determined *via* gas chromatography (GC) using a GC-2014s (Shimadzu, Kyoto, Japan) equipped with a flame ionization detector (FID). Samples for GC analysis were prepared from lyophilized cells *via* methanolysis using 15:85 v/v% sulfuric acid/methanol solution and chloroform at 100°C for 140 min.

The concentration of glucose in the culture supernatants was assayed using the Glucose CII-Test kit (Fujifilm Wako Pure Chem., Osaka, Japan). The measurement sample was prepared by diluting the culture supernatant with pure water.

The 3HBO-DEG secreted in the culture supernatant was extracted using chloroform, and the mixture was stirred for 1 h at room temperature. The chloroform was recovered from the separated layers and washed three times with an equal volume of water to remove the remaining culture medium components. After the washing, chloroform was collected and lyophilized to obtain purified 3HBO samples.

The extracted oligomer samples were dissolved in methanol and passed through a polytetrafluoroethylene (PTFE) filter before being injected into the electrospray ionization time-of-flight mass spectrometry (ESI TOF-MS) system (micrOTOFⅡ, Bruker Daltonics Co.).

## Results and Discussion

### Optimization of 3HBO-DEG Production in Batch Culture

Our previous study showed a production of 4.32 g/L of 3HBO-DEG in a shake flask culture, with 50 g/L of DEG added into the LB culture medium (Hiroe et al., 2021). To optimize the 3HBO-DEG production, batch cultures of recombinant *E. coli* BW25113Δ*adhE* harboring pGEM-*phaRC*_YB4_ *AB*, which expresses the PHA synthase gene (*phaRC*_YB4_) from *Bacillus cereus* YB-4 and the (*R*)-3-hydroxybutyryl-coenzyme A (3HB-CoA) supplier genes (*phaAB*) from *R. eutropha*, were conducted using a 250-ml mini jar fermenter (BioJr.8). The PhaRC_YB4_ used in this study is a class IV PHA synthase (Tsuge et al., 2015); it has high alcoholysis activity in addition to the CT reaction activity (Hyakutake et al., 2014) and is an enzyme capable of producing high amounts of 3HBO (Figure 1). The CT and the alcoholysis reactions both produce the oligomers terminally modified with alcohols, but the occurring phase of each reaction is different. The CT reaction occurs during the polymerization process of P(3HB), whereas the alcoholysis reaction occurs after the polymerization of P(3HB). The Δ*adhE* strain was used in this study to prevent 3HBO end modification *via* endogenous ethanol (Leonardo et al., 1993; Hiroe et al., 2013).

**FIGURE 2:**
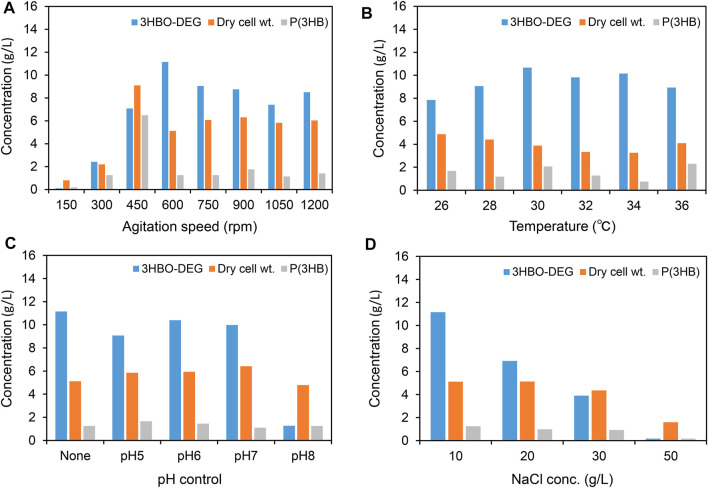
Batch cultures of recombinant *E. coli* BW25113Δ*adhE* harboring pGEM-*phaRC*_YB4_ *AB* with a 250-ml mini jar fermenter. Cells were cultured in a 100 ml LB medium supplemented with 40 g/L glucose and 50 g/L DEG for 48 h [except for **(B)**, 96 h]. The effects of the **(A)** agitation speed, **(B)** culture temperature, **(C)** pH control (pH was regulated automatically by adding 1 N NaOH or 1 N HCl for pH-stat culture), and **(D)** NaCl concentration on 3HBO-DEG secretion were examined. Unless otherwise indicated, culture conditions were 600 rpm, 1 vvm, 30°C, pH uncontrolled, and 10 g/L NaCl.

First, to examine the effect of agitation speed on 3HBO-DEG production, the agitation speed was varied in the range of 150–1,200 rpm at a constant air flow (100 ml/min). As shown in Figure 2A, the agitation speed had a definite effect on both cell growth and 3HBO-DEG production during cultivation. Improved growth was observed at agitation speeds of over 450 rpm, whereas the highest 3HBO-DEG production of 11.2 g/L was observed at 600 rpm. By contrast, P(3HB) accumulation in the cells was reduced from 71.3 wt% to 24.6 wt% by increasing the agitation speed from 450 to 600 rpm. According to the technical information of the fermenter supplier, the volumetric oxygen transfer coefficients (*k*_La_) at 450 rpm and 600 rpm were approximately 50 and 400 h^−1^, respectively. This suggestes that aerobic culture conditions promoted oligomer production rather than P(3HB) accumulation, due to the increased occurrence of CT and alcoholysis reactions. Additionally, since larger size of oligomers were remained in the cells (Hiroe et al., 2021), hydrophobic oligomers trapped in the cell membrane (Roman et al., 2020) may have been pushed out into the extracellular space by vigorous agitation. Based on the result, the agitation speed of 600 rpm was used in the subsequent cultures.

To examine the effect of culture temperature on oligomer production, the culture temperature was varied between 26 and 36°C (Figure 2B). Unless otherwise indicated, the agitation speed and air flow rate in later cultures were set at 600 rpm and 100 ml/min (1 vvm), respectively. The strain showed the highest oligomer production at 30°C; however, similar oligomer titers were obtained between 30 and 34°C. In general, PHA synthases show maximum activity at around 30°C, whereas the growth of *E. coli* is optimal at 37°C (Agus et al., 2006; Agus et al., 2010; Tomizawa et al., 2011; Mizuno et al., 2021). Therefore, a high oligomer titer was obtained at between these optimum temperatures.

Subsequently, pH-stat cultures at pH 5-8 were conducted by automatically adding 1 N NaOH and 1 N HCl, together with a pH-uncontrolled culture (Figure 2C). Among pH-stat cultures, a high titer of 10.4 g/L oligomer was obtained by culturing at pH 6, and relatively high yields were obtained between pH 5-7. However, the highest titer of 11.1 g/L oligomer was obtained under pH uncontrolled conditions. The change in pH during pH uncontrolled cultivation is described later.

In our previous study (Mizuno et al., 2021), the secretion of lactate-based oligomers was enhanced by increasing the NaCl concentration in the LB medium. Therefore, to investigate the osmotic effect of NaCl on 3HBO-DEG secretion, the NaCl concentration in the medium was varied between 10 and 50 g/L. However, the 3HBO-DEG titer and cell growth were negatively correlated with the NaCl concentration (Figure 2D). Unlike lactate-based oligomer production, the osmotic effect of NaCl had no positive impact on 3HBO-DEG production.

### Exploring Culture Medium Suitable for 3HBO-DEG Production

**FIGURE 3:**
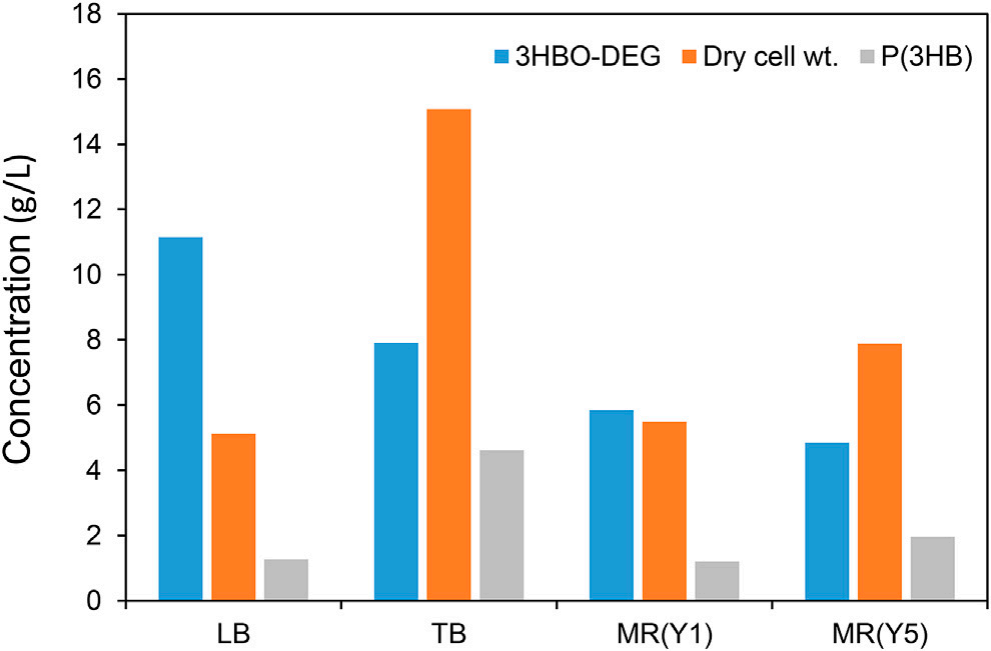
Effect of culture media on 3HBO-DEG secretion in the batch culture with a 250-ml mini jar fermenter. Recombinant *E. coli* BW25113Δ*adhE* harboring the pGEM-*phaRC*_YB4_ *AB* was cultured in a 100 ml culture medium supplemented with 40 g/L glucose and 50 g/L DEG at 30°C, 1 vvm, 600 rpm, pH uncontrolled for 48 h.

In order to explore the culture medium suitable for 3HBO-DEG production, the other three media, TB, MR (Y1), and MR (Y5), were also examined without controlling the culture pH. The TB medium is a more nutritious medium than the LB medium, whereas MR medium is a mineral medium (Wang and Lee, 1997; Kahar et al., 2005). The MR (Y1) and MR (Y5) media used in this study contained 1 g/L and 5 g/L yeast extract, respectively, because the secretion of 3HBO-DEG did not occur with the MR medium alone. The culture results are shown in Figure 3. The TB medium facilitated higher cell growth and higher P(3HB) accumulation than LB medium owing to an abundance of nutrients. However, 3HBO-DEG secretion was not enhanced, indicating that cell growth was prioritized in the TB medium. In addition, 3HBO-DEG secretion was not enhanced in the MR (Y1) and MR (Y5) media. Among the media tested, the LB medium exhibited the highest 3HBO-DEG titer and the lowest intracellular P(3HB) accumulation. To increase the yield of 3HBO-DEG, intracellular P(3HB) accumulation had to be maintained at low levels. Besides, the following experiments were carried out by using LB medium.

### Time Course of pH-Uncontrolled Batch Culture Using LB Medium

**FIGURE 4:**
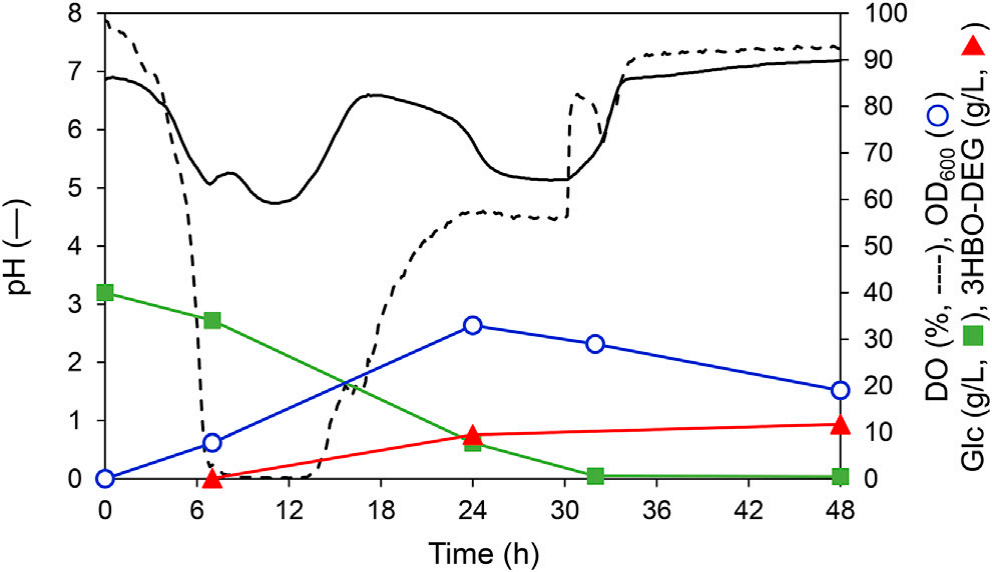
Batch culture of recombinant *E. coli* BW25113Δ*adhE* harboring pGEM-*phaRC*_YB4_ *AB* with a 250-ml mini jar fermenter. The cells were cultured in 100 ml LB medium supplemented with 40 g/L glucose and 50 g/L DEG at 30°C, 1 vvm, 600 rpm, pH uncontrolled for 48 h. Triangles denote extracellular 3HBO-DEG concentrations, squares denote the glucose concentration, circles denote cell growth (OD_600_), the solid line denotes pH, and the dashed line denotes dissolved oxygen (DO).

Optimization analysis revealed that 3HBO-DEG production could be maximized at a 600 rpm agitation speed, 1 vvm, and 30°C in the LB medium without controlling the culture pH. The time course under this culture condition is shown in Figure 4. In this culture, 40 g/L glucose was consumed in 32 h. The dissolved oxygen (DO) concentration decreased to almost zero after 6 h and began to increase after 14 h. Because the DO concentration was almost zero during 6–14 h of culturing, it is presumed that organic acids such as acetic acid, formic acid and succinic acid were generated by anaerobic metabolism (Hiroe et al., 2013; Förster and Gescher, 2014), and the pH of the culture broth decreased. In contrast, the pH increased after 14 h of culturing, which was likely caused by the consumption of these organic acids by the cells due to glucose deficiency. The culture pH changed between 4.7 and 7.2 during cultivation without pH control. For 3HBO-DEG, secretion was not observed untill 6 h of cultivation. Consequently, the 3HBO-DEG titers increased to 9.5 g/L and 11.2 g/L at 24 h and 48 h of cultivation, respectively. Further extension of culture had no effect on increasing the 3HBO-DEG titer, suggesting that oligomer production by alcoholysis in the stationary phase cells did not proceed efficiently. Therefore, culturing while maintaining CT reaction activity and alcoholysis activity of PHA synthase for a long time may lead to higher oligomer yield.

### Molecular Mass Analysis of the Secreted 3HBO-DEG

**FIGURE 5:**
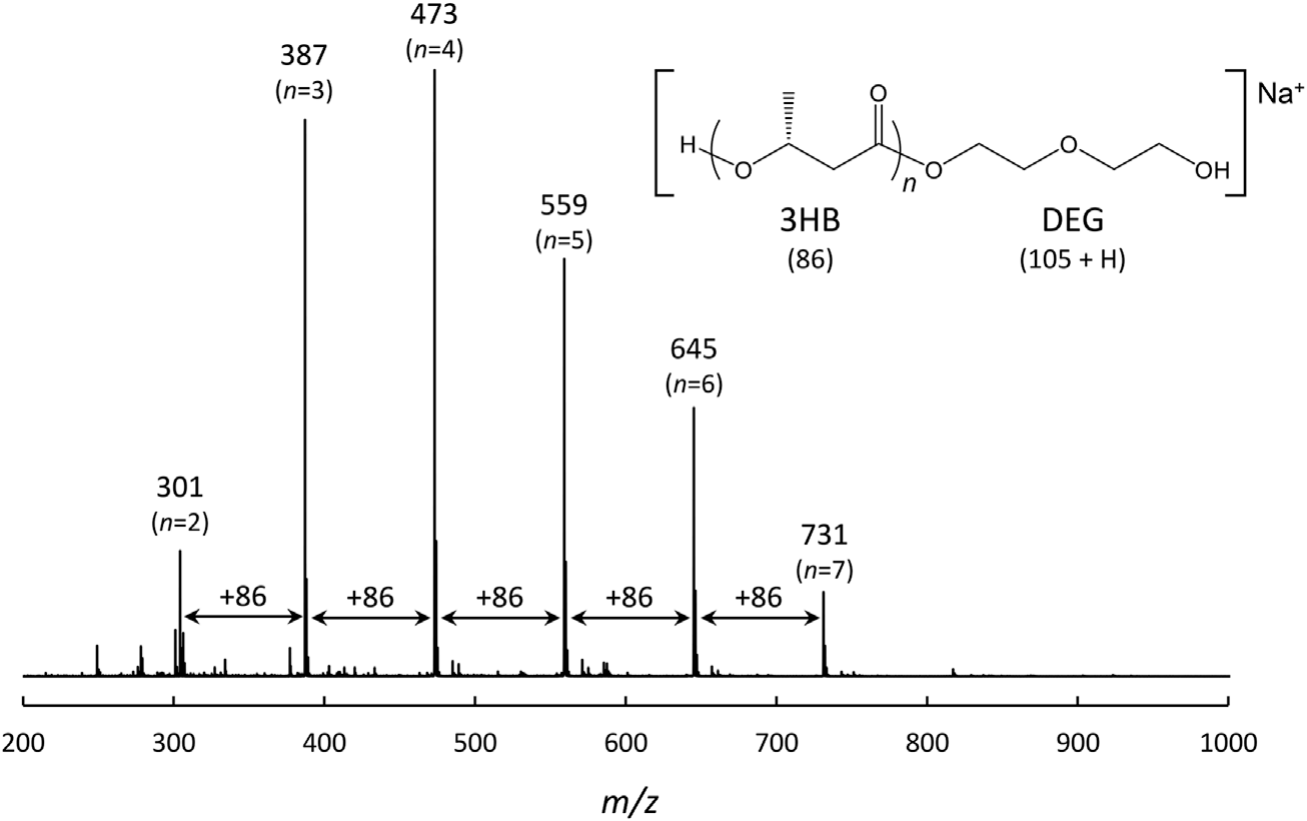
ESI TOF-MS spectrum of 3HBO-DEG produced by the batch culture of *E. coli* BW25113Δ*adhE* harboring pGEM-*phaRC*_*YB4*_ *AB* in LB medium, 30°C, 1 vvm, 600 rpm, pH uncontrolled for 48 h. ESI TOF-MS analysis was performed in the positive mode. The peak interval of *m*/*z* 86 indicates a 3HB repeating unit.

ESI TOF-MS analysis was performed to measure the molecular mass of 3HBO-DEG obtained by secretory production. Through solvent extraction with chloroform, the sample was extracted from the supernatant of the culture (Figure 4). The ESI TOF-MS spectrum is shown in Figure 5. 3HBO-DEG was detected as a Na^+^ adduct with *m*/*z* 300-800. These peaks corresponded to the molecular mass of the 2- to 7-mers 3HB terminated with DEG, thereby confirming the production of terminally modified oligomers. The molecular mass of secreted oligomers was probably regulated by the cell membrane of the host *E. coli*, which limited the size of secretion outside the cell. The molecular mass of 3HBO-DEG was consistent with that reported in our previous study (Hiroe et al., 2021). The secretory produced oligomer, which showed a high terminal modification frequency with DEG, can be preferably used as a macromonomer for synthesizing poly (ester-urethane) materials, as described previously (Utsunomia et al., 2017b; Hiroe et al., 2021; Nduko and Taguchi, 2021).

### Enhanced 3HBO-DEG Production by Fed-Batch Culture

**FIGURE 6:**
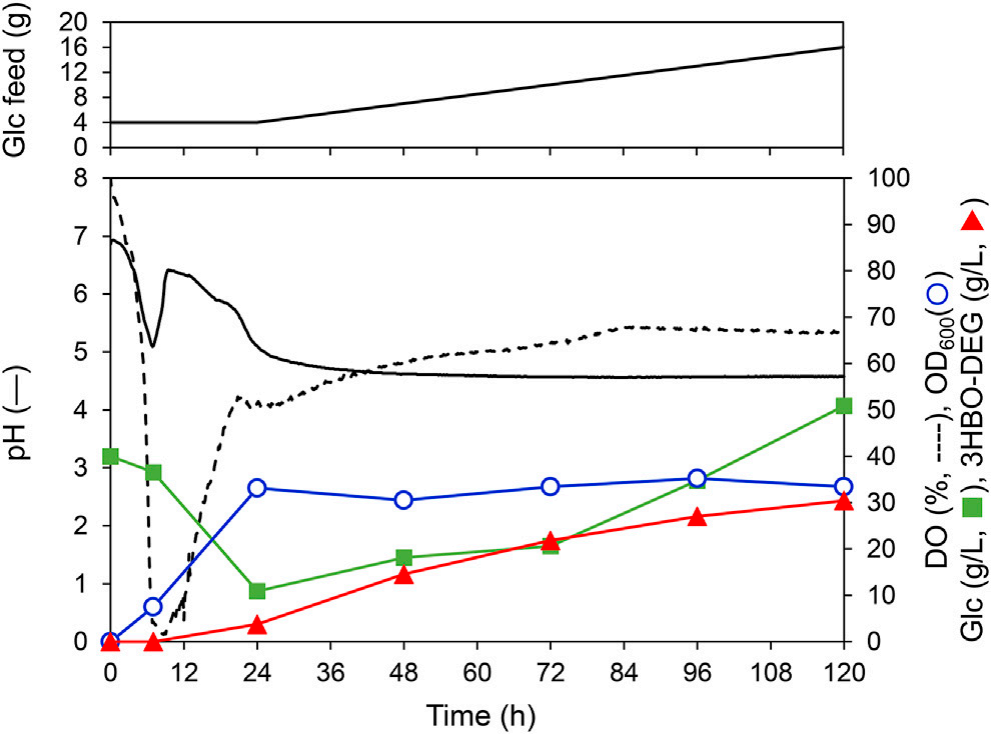
Fed-batch culture of *E. coli* BW25113Δ*adhE* harboring pGEM-*phaRC*_YB4_ *AB* with a 250-ml mini jar fermenter. Cells were cultured at 30°C, 1 vvm, 600 rpm, pH uncontrolled in LB medium (initial working volume, 100 ml) supplemented with 40 g/L glucose and 50 g/L DEG. Additionally, glucose was fed at a constant rate of 1.25 g-Glc/(L∙h) following 24 h of cultivation. Triangles denote the extracellular 3HBO-DEG concentration, squares denote the glucose concentration, circles denote cell growth (OD_600_), the solid line denotes pH, and the dashed line indicates dissolved oxygen (DO).

To further increase the 3HBO-DEG titer, fed-batch cultures using LB, TB, and MR (Y5) media were performed through feeding glucose. To this end, glucose solution was fed at 1.25 g-Glc/(L∙h) after 24 h of culturing. However, when glucose consumption by the cells was vigorous, the feed rate was temporarily increased to 2.5 g-Glc/(L∙h). Other culture conditions were the same as for the batch culture (pH uncontrolled, agitation speed 600 rpm, 1 vvm). The culture results are listed in Table 1, and the culture time course using LB medium is shown in Figure 6 as a representative example. For the LB medium, the 3HBO-DEG titer increased from 3.7 g/L to 30.4 g/L between 24 and 120 h. During this period, the culture pH was unchanged at approximately 4.5, which was a much lower value than that in the batch culture (Figure 4). In addition, DO and OD_600_ were almost unchanged at 24–120 h of culturing, thereby suggesting that the cells consumed glucose predominantly to produce oligomers. However, after 72 h of culturing, the glucose concentration in the culture broth increased owing to a decrease in glucose consumption. Finally, 11 g of the added 16 g glucose was consumed by the cells. To improve the efficiency of the oligomer yield, it would be necessary to supply glucose according to the cellular consumption.

**TABLE 1:** Secretory production of 3HBO-DEG *via E. coli* BW25113Δ*adhE* harboring pGEM-*phaRC*_YB4_ *AB*.

Operation mode	Culture equipment	Initial working vol. (ml)	Culture medium	Glc consumption (g/L)	Culture time (h)	Dry cell wt. (g/L)	P(3HB) content (wt%)	3HBO-DEG titer (g/L)^a^	3HBO-DEG yield (g/g-Glc)
Batch^b^	500-ml shake flask	100	LB	20	48	5.9	43.7	4.32	0.22
Batch^c^	250-ml mini jar	100	LB	40	48	5.1	24.6	11.2	0.28
Fed-batch^d^	250-ml mini jar	100	LB	109	120	12.4	55.0	30.4	0.28
Fed-batch	250-ml mini jar	100	TB	186	120	77.3	81.3	34.0	0.18
Fed-batch	250-ml mini jar	100	MR (Y5)	119	120	11.9	67.6	23.8	0.20
Fed-batch	2-L jar	1,000	LB	120	96	26.2	44.6	34.8	0.29

a 3HBO-DEG titer is described as volumetric weight excluding DEG.

b Data from previous study (Hiroe et al., 2021).

c Time course is shown in Figure 4.

d Time course is shown in Figure 6.

High titers of oligomer production were also observed in TB and MR (Y5) media. Specifically, the TB medium showed excellent cell growth and P(3HB)/oligomer production, with 52% of the consumed glucose converted to the intracellular P(3HB) and the secreted oligomer. In the case of LB medium, the conversion of glucose to P(3HB)/oligomer was only 37% (Table 1). For foaming during culture, TB medium tended to have more foaming than LB medium. In contrast, the MR (Y5) medium did not foam excessively. Even when utilizing the MR (Y5) medium, the relatively high 3HBO-DEG titer (23.8 g/L) was achieved on fed-batch culture. Note that P(3HB) accumulation levels were relatively high for the TB and MR (Y5) media (67.6-81.3 wt%) when compared to the LB medium (up to 55.0 wt%).

### Scaling up of 3HBO-DEG Production With a 2-L Jar Fermenter

To confirm the reproducibility of 3HBO-DEG production at a larger working volume of 1 L, a pH-uncontrolled fed-batch culture was performed using a 2-L jar fermenter. In this culture, glucose was manually fed at regular intervals, and the glucose concentration was kept within 5–20 g/L for most of the culture time. As listed in Table 1, the 3HBO-DEG titer reached 34.8 g/L after 96 h of culturing, yielding similar results to the 250-ml mini jar fermenter. Therefore, the reproducibility of 3HBO-DEG production was confirmed with a larger jar fermenter, and scale-up production was shown to be feasible.

## Conclusion

The 3HBO-DEG can be used as a macromonomer for the synthesis of PHA-based materials such as poly(3HBO-DEG-urethane); however, the high production titer of 3HBO-DEG has not been achieved. To enhance the cost-effectiveness of 3HBO-DEG production, it is necessary to increase the titer of 3HBO in the culture broth. In this study, we optimized the culture conditions for 3HBO-DEG secretory production based on 250-ml mini jar-fermenters. Among the culture conditions tested, the agitation speed had a strong effect on the secretion of 3HBO-DEG, and the highest titer was obtained at 600 rpm. The 3HBO-DEG titer was slightly higher in pH-uncontrolled cultures than in pH-stat cultures. The optimum temperature was 30°C, and the osmotic effect of NaCl had no positive impact on 3HBO-DEG production. LB and TB media showed higher titers of 3HBO-DEG than MR (Y1) and MR (Y5) media. The oligomers produced under the optimized batch culture conditions were composed of 2- to 7-mers 3HB terminated with DEG, as revealed through an ESI TOF-MS analysis. A 3HBO-DEG titer of 30.4 g/L was achieved by feeding glucose in fed-batch culture with the LB medium. Furthermore, 3HBO-DEG production of 34.8 g/L was achieved with a larger working volume of 1 L with a 2-L jar fermenter, and the reproducibility was confirmed. Thus, this study demonstrates the feasibility of the mass production of 3HBO.

## Data Availability

The original contributions presented in the study are included in the article, further inquiries can be directed to the corresponding author.
